# Molecular Mechanism of Malignant Transformation of Balb/c-3T3 Cells Induced by Long-Term Exposure to 1800 MHz Radiofrequency Electromagnetic Radiation (RF-EMR)

**DOI:** 10.3390/bioengineering9020043

**Published:** 2022-01-18

**Authors:** Zhen Ding, Xiaoyong Xiang, Jintao Li, Shuicai Wu

**Affiliations:** 1 Department of Radiation Oncology, National Cancer Center/National Clinical Research Center for Cancer/Cancer Hospital & Shenzhen Hospital, Chinese Academy of Medical Sciences and Peking Union Medical College, Shenzhen 518116, China; dingzhen0909@163.com (Z.D.); xiangxiaoyong16@163.com (X.X.); 2 Beijing Key Laboratory of Environmental and Viral Oncology, Beijing International Science and Technology Cooperation Base of Antivirus Drug, Beijing University of Technology, Beijing 100124, China; 3 College of Life Science and Bio-Engineering, Beijing University of Technology, Beijing 100124, China

**Keywords:** radiofrequency electromagnetic radiation, carcinogenic, malignant transformation, GO enrichment, KEGG pathway, lipid metabolism

## Abstract

Purpose: We aimed to investigate RF-EMR-induced cell malignant transformation. Methods: We divided Balb/c-3T3 cells into sham and expo groups. The expo groups were exposed to a 1800 MHz RF continuous wave for 40 and 60 days, for 4 h per day. The sham group was sham-exposed. Cells were harvested for a cell transformation assay, transplantation in severe combined immune deficient (SCID) mice, soft agar clone formation detection, and a transwell assay. The mRNA microarray assay was used to declare key genes and pathways. Results: The exposed Balb/c-3T3 cells showed a strong increase in cell proliferation and migration. Malignant transformation was observed in expo Balb/c-3T3 cells exposed for 40 days and 60 days, which was symbolized with visible foci and clone formation. Expo Balb/c-3T3 cells that were exposed for 40 days and 60 days produced visible tumors in the SCID mice. Lipid metabolism was the key biological process and pathway involved. The mevalonate (MVA) pathway was the key metabolic pathway. The interacted miRNAs could be further research targets to examine the molecular mechanism of the carcinogenic effects of long-term exposure. Conclusion: Exposure for 40 and 60 days to 1800 MHz RF-EMR induced malignant transformation in Balb/c-3T3 cells at the SAR of 8.0 W/kg. We declared that lipid metabolism was the pivotal biological process and pathway. The MVA pathway was the key metabolic pathway.

## 1. Introduction

The use of wireless digital technology has grown rapidly during the last couple of decades. An international action plan for the development of 5G networks has started, with a forthcoming increment in devices and density of small cells and future use of millimeter waves [1]. There is an increasing concern about radiofrequency electromagnetic radiation (RF-EMR) exposure.

No consistent conclusion had been reached on whether the RF-EMR exposure causes or promotes cancers based on epidemiological results. Nilsson et al. [2] found no evidence of an increased incidence of gliomas in recent years in Australia and Sweden. However, evidences accumulated that excessive exposure to mobile-phone frequencies over long periods of time increases the risk of brain cancer in humans. Miller’s [3] study on cell-phone exposure and glioma and acoustic neuroma, demonstrated significantly elevated risks that tended to increase with increasing latency. Moreover, the incidence of brain tumors is increasing according to the report of the Central Brain Tumor Registry of the U.S. (CBTRUS) [4]. There was a significant increase in incidence of radiographically diagnosed tumors of the pituitary from 2006 to 2012 (APC = 7.3% [95% CI: 4.1%, 10.5%]) [5]. The results of Philips A et al. [6] suggested that the environmental factor might be associated with a rise in the incidence of glioblastoma multiforme in England. The increasing evidence on cancer risks from RF radiation has had little or no effect on preventive measurements. This is due to scientific disagreements and controversies [7,8].

Many in vitro and in vivo experimental studies were executed to explore the association between RF-EMR and tumor production. In vivo studies suggested that EMR triggered cell apoptosis by regulating the caspase-3 signaling pathway [9,10] and Bcl-2 and bax gene expression [11]. Li Y et al. [12] found that apoptosis could be induced by increasing endoplasmic reticulum stress after exposure. RF-EMR depressed cell proliferation [13,14] and lead cell cycle arrest, with an increased proportion of cells at the S phase [15,16]. Moreover, RF-EMR triggered cells oxidative stress [17,18], γH2Ax foci formation, and DNA damage [19,20,21,22]. The study of the National Toxicology Program (NTP) on cell-phone RF radiation and cancer suggested an increased incidence of glioma in the brains and malignant schwannoma in the hearts of rats when they were exposed to RF-EMR [23]. The biological mechanism by which RF-EMF exposure might cause or promote cancer remains unknown. More studies are needed to further investigate and declare the potential carcinogenic effects of RF-EMR.

We aimed to declare the carcinogenic risk of long-term exposure to 1800 MHz RF-EMR, and to explore the underlying molecular mechanism. To minimize the number of passages of Balb/c-3T3 cells, we set the SAR to 8.0 W/kg to simulate the accelerated malignant transformation induced by RF-EMR exposure. Balb/c-3T3 cells were divided into sham and two expo (40-day expo and 60-day expo) groups. The sham group was sham-exposed, while the two expo groups were exposed to 1800 MHz RF-EMR for 4 h per day at the average specific absorption rate (SAR) of 8.0 W/kg, for 40 days and 60 days, respectively. All the cells were harvested for a cell transformation assay, transplantation in SCID mice, and a transwell assay. The mRNA microarray was used to declare the key genes and signaling pathways involved.

## 2. Materials and Methods

### 2.1. sXc-1800 Exposure System

**Figure 1:**
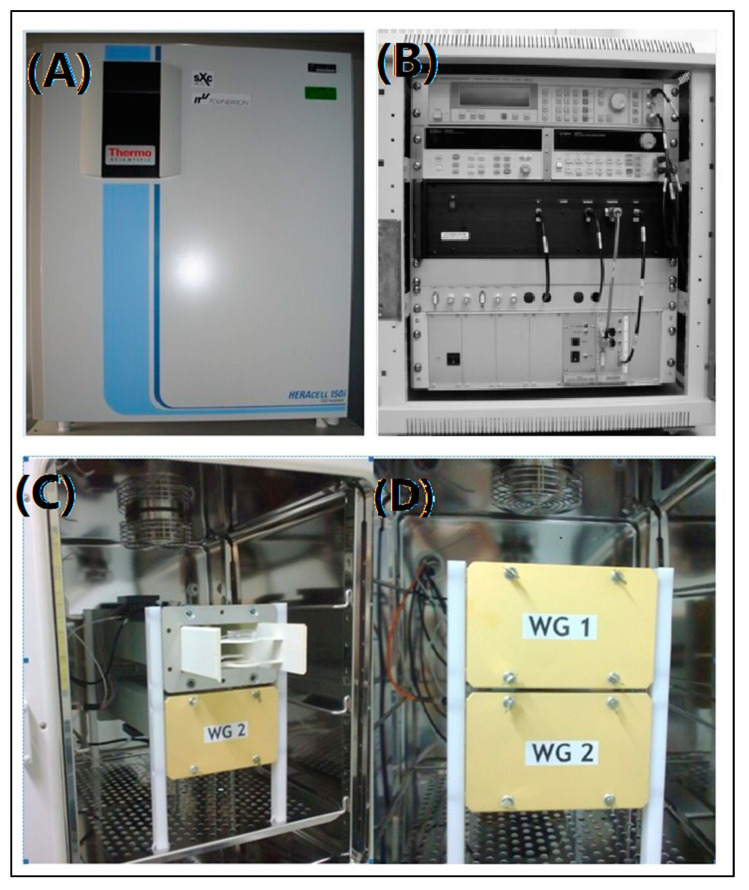
The sXc 1800 MHz exposure system. (**A**) The sXc 1800 MHz exposure system; (**B**) signal unit; (**C**) cell dish holder; (**D**) two waveguides.

The sXc-1800 exposure system (IT’IS Foundation, Zurich, Switzerland) (Figure 1), was designed to study RF-EMR, non-heating biological effects emitted from mobile communication devices. The RF setups consisted of two waveguides embeded in an incubator (Figure 1A), a signal unit (Figure 1B) and a controlling system (the PC). Cells were cultured in the standard 35 mm Petri NUNC (Nunc, Roskilde, Denmark) dishes. The standard Petri dishes were placed in the specific holder and exposed in the waveguides (Figure 1C,D). For monolayer cells, the dishes were in the H-field maximum of a standing wave in order to meet the required SAR value. In order to ensure stable exposure, H-field sensors controlled the incident field. The signal unit contained a pulse RF signal generator, RF power amplifier, function generator, data acquisition and a control unit. Much care was taken to avoid the artifacts due to temperature differences between the exposed and sham-exposed cells. Each waveguide was equipped with strong fans for rapid environmental atmospheric exchange. The airflow temperature was monitored with highly accurate DIN 1/10 Pt 100 probes fixed on top of the fan. Furthermore, the temperature response of the medium was assessed in terms of the incident field strength, liquid height, and airflow. There was a warning if the temperature difference exceeded 0.1 °C. Thus, we aimed to investigate the non-thermal effect of pulse RF-EMR.

### 2.2. Cell Culture and Exposure Protocol

The Balb/c-3T3 fibroblasts were cultured in DMEM medium, supplemented with 10% fetal bovine serum (Hyclone, Thermo Scientific, Logan City, UT, USA) and 1.0% penicillin (Hyclone), and were incubated at 37 °C and 5.0% CO_2_.

The monolayer Balb/c-3T3 cells were cultured in a 35 mm petri dish (Nunc, Roskilde, Denmark) in a concentration of 1 × 10^4^ per mL. Cell passaging was performed when the cells reached 80–90% confluency. The cells were cultured in fresh DMEM at the concentration of 1 × 10^4^ per mL. It is important to note that the sham and expo cells should be passaged at the same time. To acquire the unified and repeatable exposure environment, the DMEM medium was 3.0 mL. The malignant transformation of normal cells was a multilevel and intricate process with a long time. To minimize the number of passages of Balb/c-3T3 cells, we set the SAR to 8.0 W/kg to simulate the accelerated malignant transformation induced by RF-EMR exposure. A continuous wave was used in this protocol. The sham group was sham-exposed and incubated in the waveguide. The 40-day and 60-day expo groups were exposed for 40 and 60 days, respectively. The experiments were performed in triplicate with triplicate samples. The experiment and data analysis were carried out blind.

### 2.3. Cell Transformation Assay

In vitro cell transformation assays (CTAs) are a promising alternative to be considered in an integrated approach to carcinogen identification. After exposure, both sham and expo Balb/c-3T3 cells were seeded in T25 cell culture flasks at a concentration of 1 × 10^4^ per flask and then incubated for 30 days. The sham and expo Balb/c-3T3 cells were cultured in a medium mixed with 0.1 μg/mL TPA (12-*O*-tetradecanoylphorbol 13-acetate) (Sigma Aldrich, Saint Louis, MO, USA) for 24 h on day 1, 4, and 7. The cells were cultured in a medium on the other 27 days. Visible clone foci were counted after 30 days.

### 2.4. Transwell Assay

The sham and expo Balb/c-3T3 cells were collected and incubated with the “serum-free” medium for 24 h before the transwell assay. The cells were suspended in a serum-free DMEM medium at 1 × 10^6^ cells/mL. A volume of 600 μL DMEM containing 20% FBS was added into the basolateral chamber and a 200 μL cell suspension was seeded in the apical chamber. The plate was incubated for 24 h at 37 °C. The transwell insert was wiped and washed by PBS. After methanol fixation, the transwell insert was stained with 0.1% crystal violet.

### 2.5. Cell Transplantation in SCID Mice

Sham and expo Balb/c-3T3 cells were collected and suspended at 1 × 10^7^ cells/mL in the fresh DMEM medium. A 200 μL cell suspension was injected subcutaneously into the right front leg of an SCID mouse. All the mice were housed in a specific pathogen free (SPF) experimental animal room for 4 weeks. The HeLa cells were used as a positive control group. The mice were sacrificed to obtain the visible tumors. The size and weight of the tumors were recorded. The tumor section was stained by HE staining.

### 2.6. Soft Agar Clone Formation

The sterilized 1.2% and 0.7% agarose (Gene Company, Hongkong, China) were prepared and stored at 4 °C. A volume of 1.5 mL 1.2% agarose mixed with 1.5 mL DMEM medium with 20% FBS was slowly poured into a 6-well plate. After the base agar was solidified, 1.0 mL 0.7% agarose mixed with 1.0 mL cell suspension (5 × 10^3^ cells/mL) was plated softly over the base agar. The 6-well plate was incubated for 4 weeks at 37 °C until clones could be seen using a microscope. We used HeLa cells as a positive control.

### 2.7. mRNA Microarray Analysis

After exposure, the cells in the sham and expo groups were collected and stored with Trizol (TRIzol, Invitrogen, Carlsbad, CA, USA) agent at −80 °C. First-/second-strand cDNA was synthesized with 200 ng/μL of purified total RNA. Labeled complementary RNA (cRNA) was synthesized and amplified by in vitro transcription using the second-strand cDNA template using T7 RNA polymerase. The cRNA was fragmented and hybridized to a Mouse WG-6 V2.0 expression bead chip (Illumina, CA, USA). The results were analyzed by Genomestudio software (Illumina, CA, USA).

### 2.8. Gene Ontology (GO), Reactome, and KEGG (Kyoto Encyclopedia of Genes and Genomes) Pathway Enrichment Analysis

We employed the enrichment theory of GO terms and KEGG pathways to analyze the biological significance of expressed genes. The GO enrichment score is defined as the −log10 of the hypergeometric test *p*-value, which can be calculated by

(1) $$S_{\mathrm{GO}}=-\log_{10}\left(\overset{n}{\underset{k=m}{\sum }}\frac{\left(\frac{M}{m}\right)\left(\frac{N-M}{n-m}\right)}{\left(\frac{N}{n}\right)}\right)$$

where N, M, n, and m are the total number of proteins, the number of proteins that are annotated to the GO term, the number of proteins, and the number of proteins both in the GO term and proteins, respectively. The biological significance of expressed genes was explored by GO term enrichment analysis, including the biological process, cellular component, and molecular function using the DAVID informatics database (https://david.ncifcrf.gov (accessed on 28 December 2020)).

The KEGG pathway enrichment was similar to the definition of the GO enrichment, which is calculated by

(2) $$S_{\mathrm{KEGG}}=-\log_{10}\left(\overset{n}{\underset{k=m}{\sum }}\frac{\left(\frac{M}{m}\right)\left(\frac{N-M}{n-m}\right)}{\left(\frac{N}{n}\right)}\right)$$

where the meanings of N and n are the same as those in Equation (1). KEGG pathway enrichment was performed by the KEGG (Kyoto Encyclopedia of Genes and Genomes) database (https://www.kegg.jp (accessed on 28 December 2020)) to find the critical signal transduction pathways that these significantly expressed genes involved.

### 2.9. Protein–Protein Interaction (PPI) Network Analysis

The PPI network helped us identify the key protein–protein interaction network in the carcinogenic effects of 1800 MHz RF-EMR. PPI information of significantly expressed genes was acquired by the STRING database (https://www.string-db.org (accessed on 20 April 2021)). Moreover, the miRNAs that interacted with key genes were analyzed by the miRTarBase database (http://mirtarbase.mbc.nctu.edu.tw (accessed on 2 July 2021)).

### 2.10. Statistical Analysis

The experiments were performed in triplicate with triplicate samples. The data analysis was performed using SPSS software (Version 18). Statistical significance was assessed using the Student *t*-test where *p* < 0.05 was defined to be a significant difference.

## 3. Results

### 3.1. Malignant Transformation of Balb/c-3T3 Cells Induced by 1800 MHzRF-EMR

**Figure 2:**
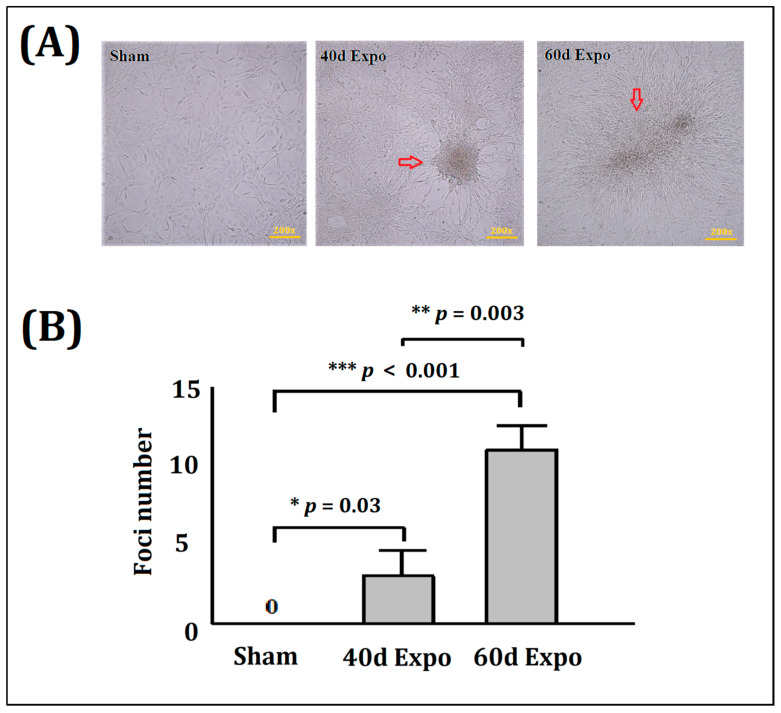
Malignant transformation of Balb/c-3T3 cells induced by 1800 MHz RF-EMR. No transformed foci were seen in sham Balb/c-3T3 cells. Visible foci (red arrows) could be observed in the 40-day and 60-day expo groups. (**A**) Foci were observed in the 40-day and 60-day expo Balb/c-3T3 cells; (**B**) histogram showing the mean foci numbers of the sham group and 40-day and 60-day expo groups. The statistical analysis was done using the *t* test. * *p* values < 0.05, ** *p* values < 0.01 and *** *p* values < 0.001.

No foci were found in the sham Balb/c-3T3 cells (Figure 2A). Visible foci were observed in the 40-day and 60-day expo groups (Figure 2A). This indicated that 1800 MHz RF-EMR exposure promoted significant malignant transformation in the Balb/c-3T3 cells. The histogram showed the mean foci numbers in the sham group and the 40-day and 60-day expo groups (Figure 2B).

### 3.2. 1800 MHz EMR Enhances Balb/c-3T3 Cells’ Ability to Migrate

**Figure 3:**
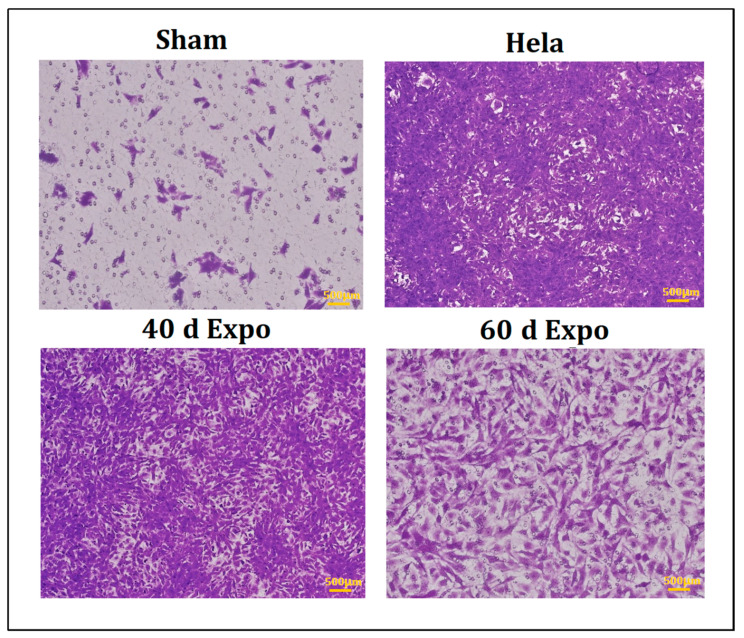
Transwell assay of Balb/c-3T3 cells after exposure. The 40-day expo and 60-day Balb/c-3T3 cells showed strong migration ability after 24 h incubation. The HeLa cells were used as the positive control group.

No significant cell proliferation was found in cells in the sham group after 24 h. As shown in Figure 3, 40-day expo and 60-day expo Balb/c-3T3 cells showed significant migration. The 40-day expo Balb/c-3T3 cells showed an enhanced migratory ability compared to the 60-day expo cells.

### 3.3. The 40-Day and 60-Day Exposed Balb/c-3T3 Cells Were Able to Form Tumors in SCID Mice

**Figure 4:**
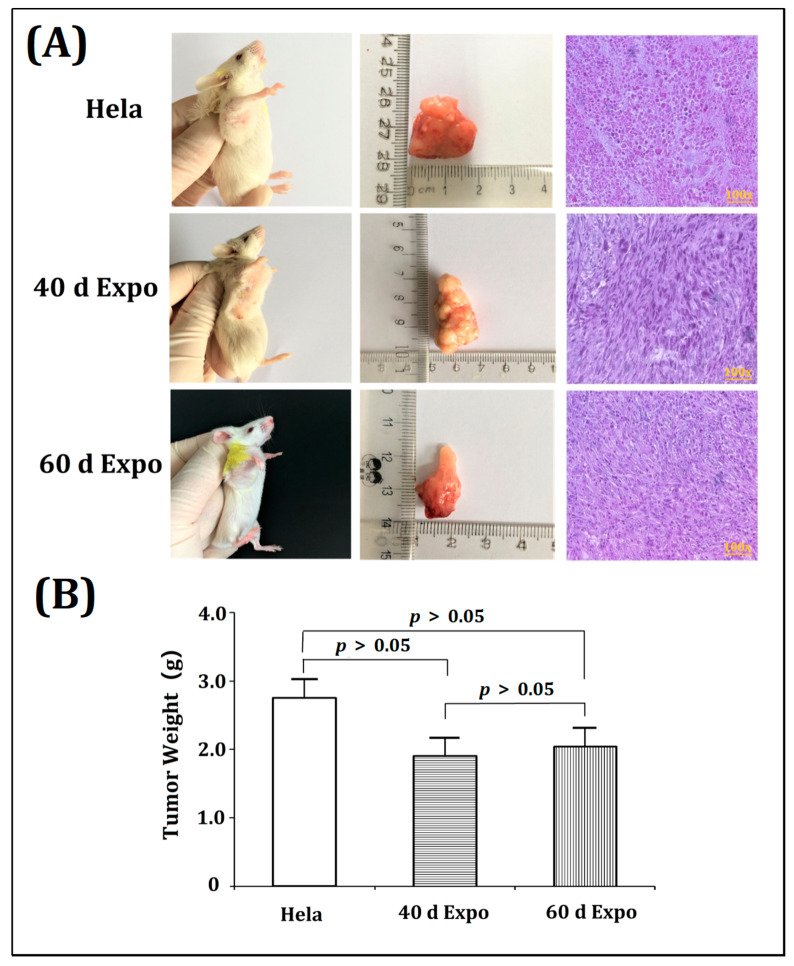
Significant tumorigenicity induced by 1800 MHz EMR in Balb/c-3T3 cells. Visible tumors were observed in SCID mice transplanted with 40-day expo and 60-day expo Balb/c-3T3 cells. The tumors were obtained and subjected to HE staining. (**A**) SCID mice were transplanted with HeLa, 40-day expo, and 60-day expo Balb/c-3T3 cells. The tumors were sectioned and subjected to HE staining. (**B**) Histogram showing the mean tumor weight obtained from SCID mice injected with HeLa, 40-day expo, and 60-day expo Balb/c-3T3 cells.

After 4 weeks of being housed in a specific pathogen free (SPF) experimental animal room, visible tumors were observed in SCID mice transplanted with 40-day expo and 60-day expo Balb/c-3T3 cells (Figure 4A). No tumors were seen in mice with sham cells. The SCID mice were then sacrificed to obtain the tumors. Figure 4B shows the mean tumor weight. Tumors were sectioned and subjected to HE staining. The 1800 MHz RF-EMR induced significant carcinogenicity in Balb/c-3T3 cells after exposure for 40 days and 60 days.

### 3.4. Clones Were Observed in 40-Day and 60-Day expo Balb/c-3T3 Cells

**Figure 5:**
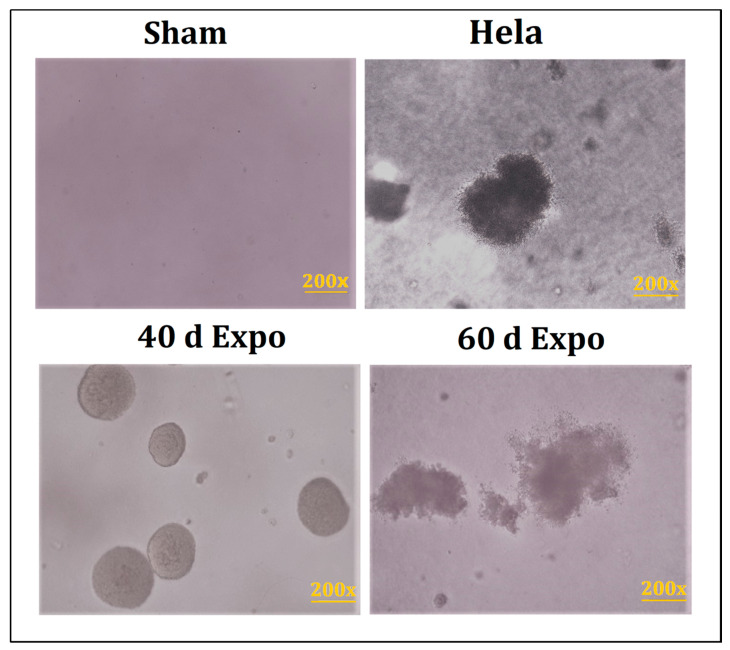
Representative images of clone formation in soft agar. Visible clones were observed in 40-day and 60-day expo Balb/c-3T3 cells. No clones were found in sham cells. HeLa cells were used as the positive control.

We seeded all the Balb/c-3T3 cells in soft agar and incubated them for 4 weeks. Visible clones were observed in 40-day expo and 60-day Balb/c-3T3 cells (Figure 5). No clones were found in sham cells. It could be manifested that 1800 MHz RF-EMR induced abnormal growth in the Balb/c-3T3 cells.

### 3.5. Significantly Expressed Genes Were Found by mRNA Microarray Detection

After exposure, the sham, 40-day expo, and 60-day expo Balb/c-3T3 cells were collected for mRNA microarray detection. There were 624 up-regulated genes and 655 down-regulated genes (fold change ≥ 2) in the 40-day expo Balb/c-3T3 cells. There were 679 up-regulated genes and 2070 down-regulated genes in the 60-day expo Balb/c-3T3 cells (fold change ≥ 2).

### 3.6. GO Term Enrichment Analysis of Significantly Expressed Genes (Fold Change ≥ 5)

**Figure 6:**
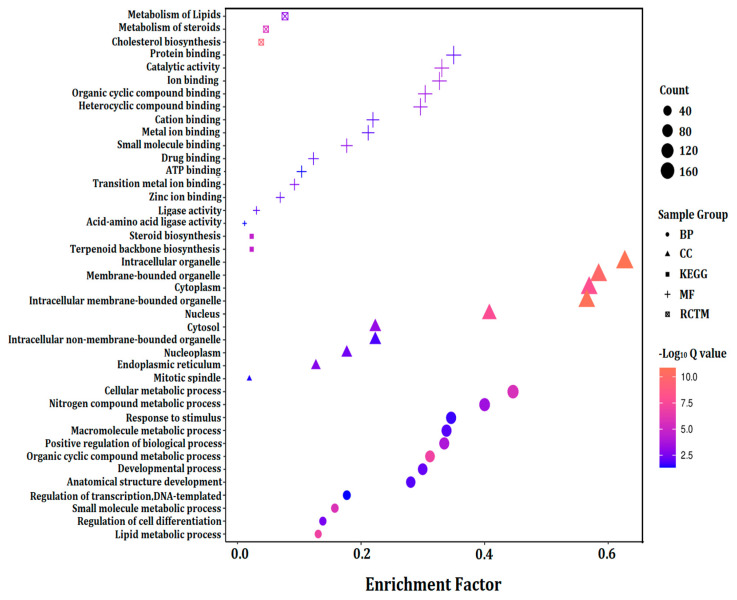
Enrichment of GO, KEGG, and Reactome of significantly expressed genes with fold change ≥ 5. The significantly expressed genes (fold change ≥ 5) in 40-day expo and 60-day expo Balb/c-3T3 cells were analyzed by gene ontology (GO), KEGG, and Reactome pathways. BP: biological process; CC: cellular component; MF: molecular function; KEGG: Kyoto encyclopedia of genes and genomes pathway; RCTM: Reactome protein.

The significantly expressed genes (fold change ≥ 5) in the 40-day expo and 60-day expo Balb/c-3T3 cells were analyzed by gene ontology (GO), KEGG, and protein–protein network analysis. Figure 6 shows the histogram of gene ontology (GO), KEGG, and the protein–protein network, including the biological process (BP), cellular component (CC), molecular function (MF), KEGG pathway, and Reactome proteins. In reference to the BP, the significantly expressed genes were enriched in the nitrogen compound, macromolecule, organic cyclic compound lipid metabolic process, and anatomical structure development. In reference to the CC, the significantly expressed genes were enriched in the intracellular and membrane-bound organelle, cytoplasm, nucleus, cytosol, nucleoplasm, endoplasmic reticulum, and the mitotic spindle. In reference to the MF, the significantly expressed genes were enriched in binding, including protein, ion, cyclic compound, cation, metal, drug, ATP, and zinc ion binding. In reference to pathways, the significantly expressed genes were enriched in steroid and terpenoid backbone biosynthesis. The Reactome pathways included cholesterol biosynthesis and metabolism of steroids and lipids.

### 3.7. Protein–Protein Interaction (PPI) Network Analysis

**Figure 7:**
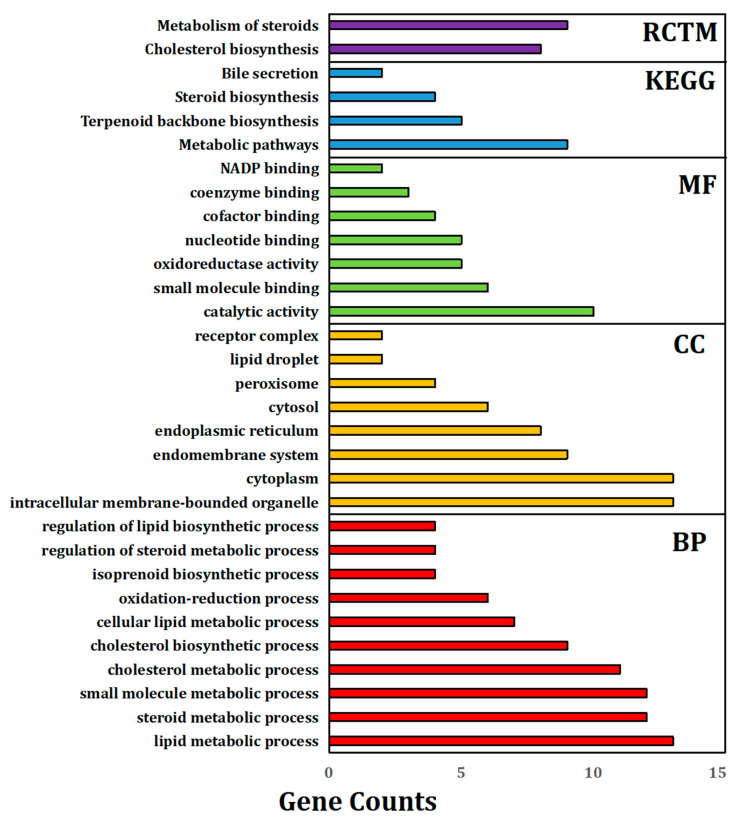
Histogram showing the pivotal genes enriched in GO, KEGG, and Reactome pathways. BP: biological process; CC: cellular component; MF: molecular function; KEGG: Kyoto encyclopedia of genes and genomes pathway; RCTM: Reactome protein.

The PPI network can help us identify the pivotal genes involved. The significantly expressed genes (fold change ≥ 5) in 40-day expo and 60-day expo Balb/c-3T3 cells were analyzed using the STRING database. The major biological processes included lipid, steroid, cholesterol, and isoprenoid metabolic processes (Figure 7). The major pathways, including the KEGG and RCTM pathways (Figure 7), were terpenoid backbone, cholesterol, and steroid biosynthesis. It could be assumed that lipid metabolism was the crucial biological process and pathway involved.

We further declared that the miRNAs interacted with the pivotal genes. In our research, we found that miRNA-124-3, miRNA-758, miRNA-362, miRNA-17, miRNA-149, miRNA-9-1, miRNA-297a, miRNA-136, miRNA-301b, miRNA-29, miRNA-223, miRNA-19b, and miRNA-15a may have participated in the regulation.

## 4. Discussion

With the rapid increase in applications of wireless devices, there are increasing concerns about exposure to radiofrequency electromagnetic radiation (RF-EMR). Previous research declared the biological impacts of exposure to RF-EMR, mainly on short-term results. Results of long-term exposure seem insufficient for a long period of research. So far, the association of exposure to RF-EMR and tumor risks is mainly based on epidemiology research. Whether the exposure to RF-EMR is carcinogenic is still inconclusive. Some epidemiological studies suggested that RF-EMR exposure might be associated with increased brain tumors, especially glioma [24]. Smith-Roe SL et al. [25] found that exposure to RFR was associated with an increase in DNA damage. A recent in vivo study of the National Toxicology Program in the US found low incidences of malignant gliomas in the brain and schwannomas in the heart of male rats exposed to RF-EMR of the two types (Code Division Multiple Access (CDMA) and Global System for Mobile Communications (GSM)) currently used in U.S. wireless networks. These findings appear to support the conclusions of the International Agency for Research on Cancer (IARC) regarding the possible carcinogenic potential of EMR. The study of Falcioni L et al. [26] on far-field exposure to RFR were consistent with and reinforce the results of the NTP study on near-field exposure, as both reported an increase in the incidence of tumors of the brain and heart in RFR-exposed Sprague–Dawley rats.

Carcinogenesis, also called oncogenesis or tumorigenesis, which involves the formation of cancer, is a complicated and multi-step process consisting of the stages of initiation, promotion, and progression. An enormous challenge for in vitro cell experiments is the long experiment period. To declare the accelerated molecular mechanism of potential carcinogenic effects of 1800 MHz RF-EMR, we set the Balb/c-3T3 cells exposed to 1800 MHz RF-EMR to an average SAR of 8.0 W/kg. In the cell transformation assay, visible foci were found in 40-day expo and 60-day expo groups under the cancer promoter’s catalysis (TPA). Cancer promoters do not affect normal cells; the process of malignant transformation is accelerated under the catalysis of cancer promoters only when malignant transformation occurs. Thus, 1800 MHz RF-EMR might act as a potential initiator of carcinogenesis, and induce significant malignant transformation of Balb/c-3T3 cells after exposure for 40 days and 60 days. Cell invasion and migration are important during the progress of tumor metastasis [27]. Tumor cells have a higher proliferation and migration capability. The 40-day expo and 60-day expo Balb/c-3T3 cells showed an evaluated proliferation and migration ability in the transwell assay compared to the sham groups. In this study, we found that NSDHL and Dhcr21 were significantly expressed in the expo groups, which had a crucial role in regulating survival, proliferation, cell cycle, migration, and invasion of breast cancer cells and promotion of breast cancer progression and metastasis [28]. Yoon SH [28] found that NSDHL knockdown in BT-20 and MDA-MB-231 resulted in a significant decrease in their viability, colony formation, migration, and invasion abilities (*p* < 0.05). Xiao Y [29] found that the over expression of NSDHL in gastric cancer (GC) was significantly correlated with local tumor invasion. Thus, exposure to 1800 MHz RF-EMR promoted cell migration by regulation of NSDHL and Dhcr21.

In the transplantations in the SCID mice experiments, visible tumors were found in SCID mice with 40-day expo and 60-day expo Balb/c-3T3 cells, indicating that 1800 MHZ RF-EMR induced Balb/c-3T3 cells’ malignant transformation when the Balb/c-3T3 cells were exposed to 1800 MHz RF-EMR for 40 and 60 days at a SAR of 8.0 W/kg. These exposed Balb/c-3T3 cells led SCID mice to have visible tumor formation.

To further study the molecular mechanism, the GO enrichment results indicated that lipid metabolism was the crucial biological process involved, including lipid, steroid, cholesterol biosynthetic, and metabolic processes. In reference to CC, the significantly expressed genes were enriched in the intracellular and membrane-bound organelle, cytoplasm, nucleus, cytosol, nucleoplasm, endoplasmic reticulum, and the mitotic spindle. The endoplasmic reticulum (ER) is a major site of protein synthesis. It is also a bulk membrane lipid biogenesis site, which occurs in the endomembrane compartment that includes the ER and Golgi apparatus [30,31,32]. In reference to pathways, the significantly expressed genes were enriched in steroid and terpenoid backbone biosynthesis. The Reactome pathways included cholesterol biosynthesis and metabolism of steroids and lipids. It was not hard to find that the lipid metabolism pathway played an important role in cell malignant transformation.

Previous research revealed that three classical lipids, fatty acids, phospholipids, and cholesterol, were dramatically increased and actively biosynthesized in cancer cells and tumors [33,34,35,36,37]. Evidence has accumulated that fatty acid synthase expression and activity are extremely low in nearly all nonmalignant adult tissues [38]. In contrast, it is significantly up-regulated in several solid and aggressive cancers [38]. The expression of choline kinase [39], a crucial enzyme in the biosynthesis of phosphatidylcholine, is up-regulated in various cancer cell lines and tumors. Recently, more and more data supported speculation that the levels of cellular cholesterol were significantly increased in cancer cells and tissues, and that cholesterol promotes cell proliferation, tumor progression, and drug resistance [40].

**Figure 8:**
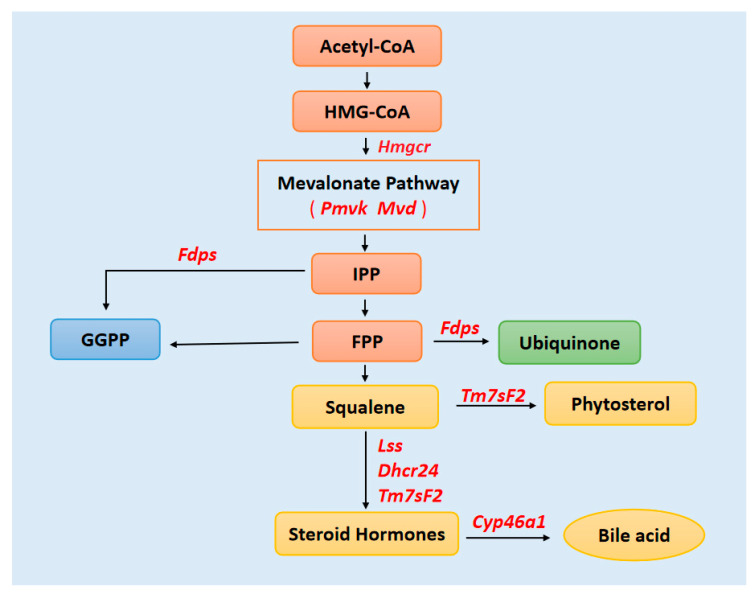
Key genes that might regulate the lipid metabolism by the mevalonate (MVA) pathway. The end product of glycolysis, acetyl-CoA, is metabolized through several enzymatic steps to cholesterol, ubiquinone, FPP, and GGPP. Key genes in tumorigenesis are highlighted in bold. IPP: isopentenyl pyrophosphate; FPP: farnesyl pyrophosphate; GGPP: Geranylgeranyl pyrophosphate.

We analyzed how the key modulators in lipid metabolic pathways are regulated. We found that the mevalonate (MVA) pathway was the key metabolic pathway that used acetyl-CoA to produce sterols and isoprenoids that are integral to tumor growth and progression (Figure 8). Chushi L et al. [41] found that HMGCR had tumor-promoting effects in gastric cancer and suggested HMGCR as a promising therapeutic target. PMVK and MVD were the key mevalonate diphosphate decarboxylase enzymes to produce isopentenyl diphosphate (IPP) [42]. Seshacharyulu P [43] found that farnesyl-diphosphate synthase (FDPS) cooperated with PTEN loss to promote prostate cancer progression through modulation of small GTPases/AKT axis. Moreover, FDPS is the key enzyme in the ubiquinone pathway. Wu J [44] found that 3β-hydroxysteroid-Δ24 reductase (DHCR24), a crucial enzyme of cholesterol biosynthetic pathway, was involved in lipid raft formation. DHCR24-mediated cholesterol metabolism might be an effective therapeutic strategy in hepatocellular carcinomas (HCCs). Futhermore, Tm7ssF2, Lss, Dhcr24, and Cyp46a1 were involved in major lipid metabolism pathways, included steroid, cholesterol, and isoprenoid metabolic processes.

Moreover, we found that miRNA-124-3, miRNA-758, miRNA-362, miRNA-17, miRNA-149, miRNA-9-1, miRNA-297a, miRNA-136, miRNA-301b, miRNA-29, miRNA-223, miRNA-19b, and miRNA-15a might participate in the regulation, by interacting with these key genes. Feng CZ’s research [45] suggested the roles of miRNA-124 in the microglial response to oxidative stress. In vitro, functional experiments revealed that up-regulation of miR-758 inhibited cell proliferation, migration, and invasion [46,47]. Mi-RNAs suppressed the proliferation, manifested as down-regulation in ovarian cancer tissues and cell lines [48]. Moreover, circular RNA could act as a microRNA-149-5p sponge to promote gastric cancer progression via the AKT1/mTOR pathway [49].

In summary, 1800 MHz RF-EMR induced Balb/c-3T3 cells’ malignant transformation at a SAR of 8.0 W/kg when the cells were exposed for over 40 and 60 days, with a promoted proliferation and migration capability and tumorigenicity in SCID mice. We declare that lipid metabolism was the pivotal biological process and pathway involved. The mevalonate (MVA) pathway was the key metabolic pathway. The interacted miRNAs could be further research targets in order to explore the molecular mechanism of the carcinogenic effects of long-term exposure.

## 5. Conclusions

A 1800 MHz RF continuous wave induced Balb/c-3T3 cells’ malignant transformation at a SAR of 8.0 W/kg when the cells were exposed for over 40 and 60 days, for 4 h per day. We declared that lipid metabolism was the pivotal biological process and pathway. The mevalonate (MVA) pathway was the key metabolic pathway.

## Data Availability

The datasets used and/or analyzed during the current study are available from the corresponding author on reasonable request.
